# Parental Age and Childhood Allergy Risk

**DOI:** 10.1001/jamanetworkopen.2025.54694

**Published:** 2026-01-20

**Authors:** Kiwako Yamamoto-Hanada, Daisuke Harama, Miori Sato, Yumiko Miyaji, Kei Sakamoto, Minaho Nishizato, Limin Yang, Natsuhiko Kumasaka, Hidetoshi Mezawa, Shintaro Iwamoto, Kyongsun Pak, Tomoki Nishizawa, Kari C. Nadeau, Maki Fukami, Yukihiro Ohya

**Affiliations:** 1 Allergy Center, National Center for Child Health and Development, Tokyo, Japan; 2 Medical Support Center for the Japan Environment and Children’s Study, National Research Institute for Child Health and Development, Tokyo, Japan; 3 Division of Biostatistics, Department of Data Management, Center for Clinical Research, National Center for Child Health and Development, Tokyo, Japan; 4 Department of Clinical Biostatistics, Graduate School of Medical and Dental Sciences, Institute of Science Tokyo, Tokyo, Japan; 5 Department of Environmental Health, Harvard T.H. Chan School of Public Health, Harvard University, Boston, Massachusetts; 6 Department of Occupational and Environmental Health, Graduate School of Medical Sciences, Nagoya City University, Aichi, Japan; 7 Division of General Allergy, Bantane Hospital, Fujita Health University, Aichi, Japan

## Abstract

**Importance:**

Allergic diseases in children are influenced by gene-environment interactions. Although advanced parental age has been associated with genetic and epigenetic changes, its relationship with childhood allergy risk remains unclear.

**Objective:**

To examine the association between parental age at childbirth and the risk of allergic diseases in early childhood.

**Design, Setting, and Participants:**

This nationwide, multicenter, population-based, prospective birth cohort study used data from the Japan Environment and Children’s Study (JECS). Participants were enrolled at 15 regional centers in Japan between January 2011 and March 2014, with follow-up data collected at child ages 1, 2, and 4 years. The present analysis was conducted from July 8, 2024, to February 4, 2025. Eligible participants were singleton live births with data on parental age and allergic outcomes. Physician-diagnosed allergy outcomes were collected via parental report. House dust mite (HDM) sensitization was assessed in a subcohort.

**Main Outcomes and Measures:**

The primary outcomes were physician-diagnosed food allergy, wheeze, asthma, and eczema at ages 1, 2, and 4 years. The secondary outcome was HDM sensitization at ages 2 and 4 years. Adjusted odds ratios (ORs) were calculated using multivariable logistic regression after multiple imputation for missing values.

**Results:**

A total of 34 942 mother-child pairs were included; the mean (SD) maternal age at entry was 31.0 (4.7) years, and 17 892 mothers (51.2%) had a medical allergy history. The prevalence of food allergy at age 1 year was 6.6% (95% CI, 6.4%-6.9%), decreasing with maternal age. Compared with children of mothers aged 25 to 29 years, those of mothers aged 35 to 39 years (OR, 0.79; 95% CI, 0.70-0.90) and aged 40 years and older (OR, 0.59; 95% CI, 0.44-0.79) had lower odds of food allergy. Children of parents both aged 35 years or older had lower odds of wheezing at age 4 years (OR, 0.89; 95% CI, 0.82-0.95). HDM was assessed in 1991 children at age 2 years and 1840 children at age 4 years, and children of older mothers also had lower odds of HDM sensitization (children of mothers aged 30-34 years, OR, 0.76; 95% CI, 0.59-0.98; children of mothers aged 35-39 years, OR, 0.68; 95% CI, 0.50-0.91).

**Conclusions and Relevance:**

In this cohort study of 34 942 mother-child pairs, children of older mothers had reduced odds of food allergy, wheezing, and HDM sensitization in early childhood, suggesting that advanced maternal age may be protective against the development of allergic diseases in early childhood.

## Key Points

**Question:**

Is advanced maternal age associated with the risk of allergic diseases in early childhood?

**Findings:**

In this nationwide prospective birth cohort study of 34 942 children in Japan, children of mothers aged 35 years or older had lower odds of physician-diagnosed food allergy at age 1 year. Similar inverse associations were observed for wheezing, eczema, and house dust mite sensitization through age 4 years.

**Meaning:**

These findings suggest that advanced maternal age may be protective against the development of allergic diseases in early childhood, potentially owing to behavioral, environmental, or biological factors associated with older parenthood.

## Teaser

This cohort study examines the association between parental age at childbirth and the risk of allergic diseases in early childhood in Japan.

## Introduction

Allergic diseases are influenced by a complex interplay of genetic and environment factors, with gene-environmental interactions playing an important role. Lifestyle changes and industrialization have contributed to the global increase in allergic diseases.^1^ Furthermore, the effects of environmental exposures may be modulated by various epigenetic mechanisms, contributing to individual susceptibility and disease development.

As a worldwide health concern, the occurrence of births to older mothers and fathers is increasing worldwide. Maternal age of 35 years or older, typically defined as *advanced maternal age*, correlates with heightened risks of premature delivery and the likelihood of having offspring with congenital disorders.^2,3^ However, the association between parental age and allergic diseases remains unclear and has not been comprehensively investigated.

In humans, aging is also known to have multifaceted effects on epigenetics, resulting in the accumulation of changes such as DNA methylation and histone modifications.^4^ As paternal age increases, the risk of DNA damage in sperm rises. Furthermore, sperm from older fathers have a higher risk of variants, which may be associated with hereditary diseases or other conditions in their children. As maternal age increases, the likelihood of chromosomal abnormalities in eggs also rises. This decline in egg quality can lead to reduced fertility, higher miscarriage rates, and an increased risk of congenital abnormalities. Thus, when the father or mother is older at the time of childbirth, there is a greater likelihood of children inheriting genes with variants. This increase in genetic variants and epigenetic modifications may contribute to the development of allergic diseases. The link between parental age and allergic conditions has yet to be explored. Previous studies have primarily focused on maternal age in relation to childhood asthma,^5,6,7^ but findings have been inconsistent. Also, these inquiries have not considered the effects of paternal age, and many were undertaken in earlier periods. Given the rapidly changing societal demographics, the impact of advanced parental age on allergic disease risk requires further investigation.

We hypothesize that the advanced age at childbirth may be associated with the risk of allergic diseases in offspring. Using data from a large birth cohort, this study aims to examine the association between parental age and the development of allergic conditions in early childhood.

## Methods

### Study Design, Setting, and Participants

This general birth cohort was established as a national, multicenter, prospective observational study, the Japan Environment and Children’s Study (JECS).^8,9,10,11,12,13,14,15,16,17^ From January 2011 to March 2014, we enrolled a total of 103 060 pregnancies, including 51 897 pregnancies with participating fathers. We launched 15 Regional Centres, encompassing a wide geographic range across Japan from north to south (Hokkaido, Miyagi, Fukushima, Chiba, Kanagawa, Koshin, Toyama, Aichi, Kyoto, Osaka, Hyogo, Tottori, Kochi, Fukuoka, and South Kyushu and Okinawa). The eligibility requirements were outlined as follows: (1) currently pregnant; (2) intending to live in the study area for the foreseeable future; (3) expected delivery between August 1, 2011, and mid-2014; and (4) fluency in the Japanese language. In total, we successfully enrolled 104 062 fetal records in the JECS. The caregivers completed questionnaires about their child and family throughout the pregnancy and until the child was age 4 years. In addition, we conducted subcohort study for 5017 children^18^ to perform detailed medical examinations such as blood sampling and home-visit survey. The eligibility criteria required analyzed children to have been engaged in the JECS until the age of 4 years, must have been born as singletons, and must have been delivered at full term (≥37 weeks of gestation). Furthermore, both the child’s mother and father were required to be participants in the JECS. Children whose mothers were teenagers at the time of birth were excluded from this present study.

The registry was conducted in compliance with the Ethical Guidelines for Medical and Health Research involving Human Subjects in Japan. The JECS protocol was approved by the Ministry of the Environment’s Institutional Review Board on Epidemiological Studies and the Ethics Committees of all participating institutions. We secured written informed consent from all participants. For this study, we used the fixed dataset for age 3 years, updated on October 3, 2022, and the fixed dataset for age 4 years, updated on April 12, 2023. This study was conducted and reported in accordance with the Strengthening the Reporting of Observational Studies in Epidemiology (STROBE) reporting guidelines for cohort studies.

### Variables

Exposure variables are defined as follows. We defined parental age categories using 35 years as the threshold because, in both obstetric and epidemiological research, maternal age of 35 years or older is commonly regarded as the standard definition of advanced maternal age. This cutoff ensured clinical relevance and comparability with previous studies. Parental age at birth combinations categorized as both mother and father aged 35 years or older, mother aged 35 years or older and father aged 34 years or younger, mother aged 34 years or younger and father aged 35 years or older, and both mother and father aged 34 years or younger. Maternal age at birth categorized as 20 to 24 years, 25 to 29 years, 30 to 34 years, 35 to 39 years, and 40 years or older. Categorizing maternal age in 5-year intervals is a common approach in perinatal and pediatric epidemiology, allowing for the assessment of dose-response trends across increasing maternal age groups.

The outcomes related to allergies were evaluated using a questionnaire survey conducted by mail. In addition, blood samples were collected for IgE testing from participants in the subcohort study.^18^ Wheeze, asthma, eczema, atopic dermatitis diagnosis, rhinitis, and hay fever were defined according to the International Study of Asthma and Allergies in Childhood questionnaires.^19^ House dust mite (HDM) IgE and Japanese cedar IgE^20,21,22,23^ were measured at ages 2 and 4 years (eTable in Supplement 1). We evaluated food allergy according to the parental-reported questionnaire at 4 years of age. Food allergy diagnosis was defined as an affirmative answer from the caregiver to the question, “Has your child had a food allergy diagnosis by a medical doctor?” as previously applied at 1 and 4 years.^14,24,25^ Confounders included affiliated regional centers, maternal history of allergies, paternal history of allergies, maternal highest educational attainment, household income, child’s sex, birth weight, mode of delivery, number of siblings, household smoking during pregnancy, daycare, pets, and maternal body mass index.

### Statistical Analysis

Statistical analyses were conducted from July 8, 2024, to February 4, 2025. We included singleton, full-term–born children who participated in the survey up to age 4 years, had complete data for both maternal and paternal ages, and whose mothers were aged 20 years or older. In all maternal age models, the 25 to 29 year group was used as the reference category, as it is generally considered the optimal age for childbirth according to previous epidemiological literature. Using this group as the baseline enabled a clearer comparison of risks associated with both younger and older maternal ages.

Means and SDs were calculated for continuous variables, and numbers and proportions were computed for categorical variables. Univariable and multivariable logistic regression analyses were performed to estimate unadjusted and adjusted odds ratios (ORs) with 95% CIs for associations between parental age and childhood allergic outcomes.

All multivariable models included the following potential confounders: affiliated regional centers, maternal and paternal history of allergies, maternal educational attainment, household income, child’s sex, birth weight, mode of delivery, number of siblings, household smoking during pregnancy, daycare attendance, pet ownership, and maternal body mass index. To account for regional variation, study site was included as a categorical covariate.

To examine the potential influence of paternal age, stratified analyses were conducted according to paternal age categories (≥35 years vs ≤34 years). These models were used to assess whether associations between maternal age and allergic outcomes differed by paternal age. Results from these stratified analyses were presented alongside the main multivariable models.

Missing data were handled using multiple imputation by chained equations, and pooled estimates were obtained using Rubin rules. Given the exploratory nature of this study, *P* values were presented as descriptive indicators rather than as formal tests of statistical significance. No adjustments for multiple comparisons were applied. Analyses involving IgE levels were limited to children with available IgE data, following the same analytical procedures. All analyses were performed using R statistical software version 4.4.2 (The R Project for Statistical Computing). Further details on the imputation procedures, handling of site-level variation, and full model specifications are provided in the eMethods in Supplement 1.

## Results

The analysis included 34 942 participants up to the age of 4 years (eFigure in Supplement 1); the mean (SD) maternal age at entry was 31.0 (4.7) years, and 17 892 mothers (51.2%) reported a medical history of allergy (Table 1). Blood test results were available for 1991 children at age 2 years and 1840 children at age 4 years. The eTable in Supplement 1 illustrates the characteristics of allergic conditions.

**Table 1.** Baseline Characteristics of the Cohort

Characteristic	Participants, No. (%) (N = 34 942)
Study prefecture	
Chiba or Kanagawa	4227 (12.1)
Miyagi or Fukushima	8487 (24.3)
Hokkaido	1902 (5.4)
Koshin (Yamanashi or Shinshu)	3414 (9.8)
Aichi	1742 (5.0)
Toyama, Kyoto, Osaka or Hyogo	8035 (23.0)
Tottori or Kochi	2335 (6.7)
Fukuoka, Kumamoto, or Miyazaki	4576 (13.1)
Okinawa	224 (0.6)
Maternal age at entry, mean (SD), y	31.0 (4.7)
Maternal age group at entry, y	
20-24	2965 (8.5)
25-29	10 595 (30.3)
30-34	12 857 (36.8)
35-39	7262 (20.8)
≥40	1263 (3.6)
Paternal age at entry, mean (SD), y	33.0 (5.7)
Paternal age group at entry, y	
≤19	31 (0.1)
20-24	1826 (5.2)
25-29	8132 (23.3)
30-34	11 754 (33.6)
35-39	8828 (25.3)
≥40	4371 (12.5)
Parental age category, y	
Mother, ≤34; father, ≤34	20 157 (57.7)
Mother, ≤34; father, ≥35	6260 (17.9)
Mother, ≥35; father, ≤34	1586 (4.5)
Mother, ≥35; father, ≥35	6939 (19.9)
Maternal allergy history	
No	16 960 (48.5)
Yes	17 892 (51.2)
Missing	90 (0.3)
Paternal allergy history	
No	19 739 (56.5)
Yes	14 856 (42.5)
Missing	347 (1.0)
Highest education	
Middle school or high school	10 848 (31.0)
College of technology	8769 (25.1)
University graduate	15 086 (43.2)
Missing	239 (0.7)
Annual income, ¥^a^	
<6 000 000	23 541 (67.4)
≥6 000 000	9371 (26.8)
Missing	2030 (5.8)
Child’s sex	
Boy	17 746 (50.8)
Girl	17 196 (49.2)
Birth weight, mean (SD), g	3060.8 (364.8)
Missing	18 (<0.1)
Delivery mode	
Caesarean delivery	6098 (17.5)
Vaginal	28 767 (82.3)
Missing	77 (0.2)
Siblings	
0	17 366 (49.7)
≥1	17 576 (50.3)
Household smoking during pregnancy	
No	18 214 (52.1)
Yes	16 588 (47.5)
Missing	140 (0.4)
Pet	
No	28 534 (81.7)
Yes	6290 (18.0)
Missing	118 (0.3)
Daycare center	
No	18 005 (51.5)
Yes	16 652 (47.7)
Missing	285 (0.8)
Maternal body mass index^b^	
<25.0	29 955 (85.7)
≥25.0	4330 (12.4)
Missing	657 (1.9)

^a^ As of December 5, 2025, 1¥ = US $0.0064.

^b^ Body mass index is calculated as weight in kilograms divided by height in meters squared.

### Maternal Age and Odds of Childhood Allergy at Ages 1 and 2 Years

The prevalence of wheezing up to 1 year of age was 18.4% (95% CI, 18.0%-18.8%), and that of food allergy diagnosed by a physician was 6.6% (95% CI, 6.4%-6.9%) (eTable in Supplement 1). The prevalence of food allergy decreased with increasing maternal age: 772 of 10 595 children (7.3%) of mothers aged 25 to 29 years, 443 of 7262 children (6.1%) of mothers aged 35 to 39 years, and 54 of 1263 children (4.3%) of mothers aged 40 years or older had food allergies (Table 2). When using maternal age 25 to 29 years as the reference, children of mothers aged 20 to 24 years had higher odds of wheezing by age 1 year (OR, 1.13; 95% CI, 1.01-1.26), whereas those of mothers aged 35 to 39 years (OR, 0.89; 95% CI, 0.82-0.96) and 40 years or older (OR 0.73; 95% CI, 0.62-0.87) had lower odds. For food allergy reaction at 1 year, the odds were also lower for children of mothers aged 35 to 39 years (OR, 0.79; 95% CI, 0.70-0.90) and 40 years or older (OR, 0.59; 95% CI, 0.44-0.79).

**Table 2.** Association of Maternal Age With Childhood Allergy at Ages 1 and 2 Years^a^

Outcome and maternal age group	No. of children with outcome/total No. (%)	Univariable model		Multivariable model		Multiple imputation	
		OR (95% CI)	*P* value	OR (95% CI)	*P* value	OR (95% CI)	*P* value
Ever wheezing at age 1 y							
20-24 y	560/2949 (19.0)	1.03 (0.93-1.14)	.58	1.13 (1.01-1.27)	.04	1.13 (1.01-1.26)	.04
25-29 y	1953/10 537 (19.0)	1 [Reference]	NA	1 [Reference]	NA	1 [Reference]	NA
30-34 y	2421/12 793 (19.0)	1.03 (0.96-1.09)	.45	0.96 (0.89-1.03)	.23	0.95 (0.89-1.02)	.17
35-39 y	1314/7225 (18.0)	0.98 (0.90-1.05)	.56	0.90 (0.82-0.97)	.01	0.89 (0.82-0.96)	<.001
≥40 y	186/1258 (15.0)	0.76 (0.65-0.90)	.001	0.70 (0.58-0.84)	<.001	0.73 (0.62-0.87)	<.001
Food allergy physician’s diagnosis at age 1 y							
20-24 y	176/2965 (5.9)	0.80 (0.68-0.95)	.01	0.91 (0.76-1.09)	.33	0.90 (0.76-1.07)	.26
25-29 y	772/10 595 (7.3)	1 [Reference]	NA	1 [Reference]	NA	1 [Reference]	NA
30-34 y	875/12 857 (6.8)	0.93 (0.84-1.03)	.15	0.91 (0.83-1.02)	.11	0.90 (0.81-1.00)	.05
35-39 y	443/7262 (6.1)	0.83 (0.73-0.93)	.002	0.78 (0.68-0.89)	<.001	0.79 (0.70-0.90)	<.001
≥40 y	54/1263 (4.3)	0.57 (0.43-0.76)	<.001	0.59 (0.44-0.80)	<.001	0.59 (0.44-0.79)	<.001
Food allergy reactions at age 1 y							
20-24 y	415/2959 (14.0)	0.79 (0.70-0.89)	<.001	0.89 (0.78-1.00)	.05	0.86 (0.76-0.96)	.01
25-29 y	1816/10 576 (17.0)	NA	NA	NA	NA	NA	NA
30-34 y	2123/12 838 (17.0)	0.95 (0.90-1.02)	.20	0.96 (0.89-1.03)	.25	0.95 (0.89-1.02)	.13
35-39 y	1134/7250 (16.0)	0.90 (0.83-0.97)	.01	0.89 (0.82-0.97)	.01	0.90 (0.82-0.97)	.009
≥40 y	170/1261 (13.0)	0.75 (0.64-0.89)	.001	0.79 (0.66-0.94)	.01	0.78 (0.66-0.92)	.005
Atopic dermatitis physician’s diagnosis at age 1 y							
20-24 y	120/2965 (4.0)	1.01 (0.82-1.23)	.95	1.11 (0.88-1.39)	.38	1.05 (0.85-1.30)	.63
25-29 y	426/10 595 (4.0)	1 [Reference]	NA	1 [Reference]	NA	1 [Reference]	NA
30-34 y	538/12 857 (4.2)	1.04 (0.91-1.19)	.53	1.05 (0.91-1.21)	.45	1.01 (0.89-1.16)	.84
35-39 y	288/7262 (4.0)	0.99 (0.84-1.15)	.85	1.00 (0.84-1.17)	.96	0.95 (0.81-1.12)	.54
≥40 y	44/1263 (3.5)	0.86 (0.63-1.19)	.37	0.96 (0.69-1.34)	.82	0.88 (0.64-1.21)	.43
Eczema defined by International Study of Asthma and Allergies in Childhood at age 1 y							
20-24 y	470/2962 (16.0)	0.91 (0.82-1.02)	.12	0.97 (0.86-1.09)	.60	0.94 (0.84-1.06)	.33
25-29 y	1807/10 588 (17.0)	1 [Reference]	NA	1 [Reference]	NA	1 [Reference]	NA
30-34 y	2318/12 850 (18.0)	1.07 (1.00-1.14)	.05	1.05 (0.98-1.13)	.17	1.05 (0.98-1.13)	.14
35-39 y	1336/7257 (18.0)	1.09 (1.01-1.19)	.02	1.08 (0.99-1.17)	.08	1.08 (0.99-1.17)	.07
≥40 y	222/1263 (18.0)	1.04 (0.89-1.21)	.65	1.05 (0.89-1.23)	.57	1.06 (0.91-1.25)	.43
House dust mite IgE at age 2 y							
20-24 y	56/124 (45.0)	1.11 (0.74-1.63)	.63	1.14 (0.75-1.73)	.55	1.01 (0.67-1.52)	.96
25-29 y	215/503 (43.0)	1 [Reference]	NA	1 [Reference]	NA	1 [Reference]	NA
30-34 y	250/651 (38.0)	0.84 (0.66-1.06)	.14	0.90 (0.70-1.16)	.41	0.90 (0.7-1.14)	.37
35-39 y	173/405 (43.0)	1.00 (0.76-1.30)	.99	1.09 (0.82-1.46)	.53	1.07 (0.81-1.40)	.64
≥40 y	49/100 (49.0)	1.28 (0.84-1.97)	.25	1.30 (0.81-2.08)	.28	1.30 (0.83-2.01)	.26
Japanese cedar IgE at age 2 y							
20-24 y	11/124 (8.9)	0.90 (0.45-1.79)	.77	0.62 (0.28-1.35)	.23	0.705 (0.34-1.45)	.34
25-29 y	49/503 (9.7)	1 [Reference]	NA	1 [Reference]	NA	1 [Reference]	NA
30-34 y	57/651 (8.8)	0.89 (0.59-1.32)	.57	0.94 (0.61-1.45)	.79	0.932 (0.61-1.4)	.73
35-39 y	35/405 (8.6)	0.88 (0.55-1.38)	.57	0.85 (0.52-1.40)	.53	0.869 (0.54-1.39)	.56
≥40 y	13/100 (13.0)	1.39 (0.72-2.66)	.33	1.46 (0.73-2.97)	.28	1.35 (0.68-2.66)	.40

Abbreviations: NA, not applicable; OR, odds ratio.

^a^ Multivariable logistic regression models were constructed by including confounding factors (ie, affiliated regional centers, maternal history of allergies, paternal history of allergies, maternal highest educational attainment, household income, child’s sex, birth weight, mode of delivery, number of siblings, household smoking during pregnancy, daycare, pets, and maternal body mass index), and the adjusted (multivariable model) ORs and their CIs were calculated for each explanatory variable. For each explanatory variable, we calculated adjusted ORs from the multivariable model, as well as adjusted ORs obtained after multiple imputation of missing values, together with their 95% CIs.

### Maternal Age and Odds of Childhood Allergy at Age 4 Years

At age 4 years, the prevalence of ever wheezing was 29.2% (95% CI, 28.7%-29.7%), and that of food allergy was 5.8% (95% CI, 5.5%-6.0%) (eTable in Supplement 1). Both outcomes tended to decrease with increasing maternal age. Children of mothers aged 35 to 39 years (OR, 0.90; 95% CI, 0.84-0.97) and 40 years or older (OR, 0.85; 95% CI, 0.74-0.97) had lower odds of ever wheezing, whereas those of mothers aged 20 to 24 years had higher odds (OR, 1.14; 95% CI, 1.04-1.26) (Table 3).

**Table 3.** Association of Maternal Age With Childhood Allergy at Age 4 Years^a^

Outcome and maternal age group	No. of children with outcome/total No. (%)	Univariable model		Multivariable model		Multiple imputation	
		OR (95% CI)	*P* value	OR (95% CI)	*P* value	OR (95% CI)	*P* value
Current wheezing at age 4 y							
20-24 y	445/2953 (15.0)	1.04 (0.93-1.17)	.48	1.07 (0.94-1.22)	.29	1.08 (0.96-1.22)	.19
25-29 y	1538/10 575 (15.0)	1 [Reference]	NA	1 [Reference]	NA	1 [Reference]	NA
30-34 y	1870/12 827 (15.0)	1.00 (0.93-1.08)	.94	0.99 (0.91-1.07)	.80	0.97 (0.90-1.05)	.51
35-39 y	1084/7251 (15.0)	1.03 (0.95-1.13)	.45	1.01 (0.92-1.11)	.83	1.00 (0.91-1.09)	.95
≥40 y	170/1262 (13.0)	0.91 (0.77-1.08)	.31	0.92 (0.77-1.12)	.42	0.91 (0.77-1.08)	.32
Ever wheezing at age 4 y							
20-24 y	934/2956 (32.0)	1.08 (0.99-1.19)	.08	1.13 (1.02-1.25)	.02	1.14 (1.04-1.26)	.004
25-29 y	3165/10 576 (30.0)	1 [Reference]	NA	1 [Reference]	NA	1 [Reference]	NA
30-34 y	3722/12 830 (29.0)	0.96 (0.90-1.01)	.13	0.94 (0.89-1.00)	.05	0.93 (0.89-0.99)	.03
35-39 y	2049/7244 (28.0)	0.92 (0.86-0.99)	.02	0.91 (0.85-0.99)	.02	0.90 (0.84-0.97)	.004
≥40 y	328/1258 (26.0)	0.83 (0.73-0.94)	.005	0.84 (0.73-0.98)	.02	0.85 (0.74-0.97)	.02
Current asthma at age 4 y							
20-24 y	333/2965 (11.0)	1.14 (1.01-1.28)	.03	1.15 (1.00-1.31)	.05	1.27 (1.11-1.46)	<.001
25-29 y	961/10 595 (9.1)	1 [Reference]	NA	1 [Reference]	NA	1 [Reference]	NA
30-34 y	1046/12 857 (8.1)	0.94 (0.87-1.02)	.14	0.94 (0.86-1.03)	.17	0.88 (0.79-0.96)	.006
35-39 y	627/7262 (8.6)	0.96 (0.88-1.05)	.43	0.95 (0.86-1.05)	.30	0.92 (0.83-1.03)	.17
≥40 y	117/1263 (9.3)	0.93 (0.78-1.12)	.46	0.94 (0.77-1.15)	.56	1.03 (0.84-1.27)	.77
Ever asthma at age 4 y							
20-24 y	402/2948 (14.0)	1.14 (1.01-1.28)	.03	1.15 (1.00-1.31)	.05	1.16 (1.03-1.32)	.02
25-29 y	1285/10 562 (12.0)	1 [Reference]	NA	1 [Reference]	NA	1 [Reference]	NA
30-34 y	1479/12 819 (12.0)	0.94 (0.87-1.02)	.14	0.94 (0.86-1.03)	.17	0.92 (0.85-1.00)	.06
35-39 y	852/7237 (12.0)	0.96 (0.88-1.05)	.43	0.95 (0.86-1.05)	.30	0.94 (0.85-1.03)	.20
≥40 y	144/1258 (11.0)	0.93 (0.78-1.12)	.46	0.94 (0.77-1.15)	.56	0.94 (0.79-1.14)	.55
Current rhinitis at age 4 y							
20-24 y	271/2965 (9.1)	1.15 (1.00-1.34)	.05	1.08 (0.92-1.27)	.34	1.09 (0.94-1.27)	.22
25-29 y	849/10 595 (8.0)	1 [Reference]	NA	1 [Reference]	NA	1 [Reference]	NA
30-34 y	1037/12 857 (8.1)	1.01 (0.91-1.11)	.88	1.05 (0.95-1.17)	.30	1.06 (0.96-1.17)	.22
35-39 y	525/7262 (7.2)	0.90 (0.80-1.00)	.05	0.97 (0.86-1.11)	.68	0.98 (0.87-1.11)	.71
≥40 y	110/1263 (8.7)	1.09 (0.89-1.35)	.39	1.20 (0.96-1.51)	.12	1.22 (0.99-1.52)	.06
Hay fever at age 4 y							
20-24 y	315/2965 (11.0)	1.13 (0.99-1.28)	.08	1.06 (0.92-1.23)	.39	1.08 (0.94-1.25)	.25
25-29 y	1011/10 595 (9.5)	1 [Reference]	NA	1 [Reference]	NA	1 [Reference]	NA
30-34 y	1205/12 857 (9.4)	0.98 (0.90-1.07)	.66	1.02 (0.92-1.12)	.75	1.03 (0.94-1.13)	.55
35-39 y	661/7262 (9.1)	0.95 (0.86-1.05)	.32	1.02 (0.91-1.14)	.73	1.03 (0.93-1.15)	.55
≥40 y	123/1263 (9.7)	1.02 (0.84-1.25)	.82	1.08 (0.87-1.34)	.47	1.15 (0.93-1.40)	.19
Food allergy physician’s diagnosis at age 4 y							
20-24 y	175/2965 (5.9)	0.97 (0.81-1.15)	.69	1.05 (0.87-1.27)	.60	1.02 (0.85-1.22)	.84
25-29 y	646/10 595 (6.1)	1 [Reference]	NA	1 [Reference]	NA	1 [Reference]	NA
30-34 y	754/12 857 (5.9)	0.96 (0.86-1.07)	.45	0.96 (0.86-1.08)	.55	0.97 (0.87-1.08)	.59
35-39 y	384/7262 (5.3)	0.86 (0.76-0.98)	.02	0.88 (0.76-1.01)	.07	0.88 (0.76-1.00)	.06
≥40 y	58/1263 (4.6)	0.74 (0.57-0.98)	.03	0.82 (0.61-1.09)	.18	0.79 (0.6-1.05)	.10
Food allergy reactions at age 4 y							
20-24 y	543/2965 (18.0)	0.86 (0.77-0.95)	.004	0.94 (0.84-1.05)	.27	0.92 (0.83-1.03)	.16
25-29 y	2197/10 595 (21.0)	1 [Reference]	NA	1 [Reference]	NA	1 [Reference]	NA
30-34 y	2474/12 857 (19.0)	0.91 (0.85-0.97)	.004	0.90 (0.84-0.97)	.006	0.91 (0.85-0.98)	.007
35-39 y	1256/7262 (17.0)	0.80 (0.74-0.86)	<.001	0.79 (0.73-0.87)	<.001	0.81 (0.75-0.88)	<.001
≥40 y	189/1263 (15.0)	0.67 (0.57-0.79)	<.001	0.72 (0.60-0.85)	<.001	0.72 (0.61-0.84)	<.001
Atopic dermatitis physician’s diagnosis at age 4 y							
20-24 y	269/2965 (9.1)	1.12 (0.96-1.28)	.15	1.14 (0.98-1.34)	.10	1.15 (1.00-1.34)	.05
25-29 y	873/10 595 (8.2)	1 [Reference]	NA	1 [Reference]	NA	1 [Reference]	NA
30-34 y	1053/12 857 (8.2)	0.99 (0.90-1.09)	.89	0.96 (0.87-1.06)	.48	0.97 (0.88-1.06)	.53
35-39 y	558/7262 (7.7)	0.92 (0.83-1.03)	.18	0.91 (0.81-1.03)	.15	0.91 (0.81-1.02)	.12
≥40 y	89/1263 (7.0)	0.84 (0.67-1.06)	.14	0.88 (0.69-1.12)	.29	0.88 (0.70-1.11)	.27
Eczema defined by International Study of Asthma and Allergies in Childhood at age 4 y							
20-24 y	386/2955 (13.0)	0.98 (0.87-1.11)	.70	1.06 (0.92-1.21)	.41	1.05 (0.92-1.19)	.46
25-29 y	1410/10 572 (13.0)	1 [Reference]	NA	1 [Reference]	NA	1 [Reference]	NA
30-34 y	1774/12 839 (14.0)	1.04 (0.97-1.13)	.29	1.02 (0.94-1.11)	.62	1.01 (0.94-1.09)	.71
35-39 y	1007/7251 (14.0)	1.05 (0.96-1.14)	.29	1.04 (0.94-1.14)	.43	1.03 (0.94-1.13)	.56
≥40 y	165/1261 (13.0)	0.98 (0.82-1.16)	.80	1.03 (0.86-1.25)	.74	1.02 (0.85-1.22)	.82
House dust mite IgE at age 4 y							
20-24 y	44/124 (35.0)	1.01 (0.67-1.52)	.95	1.04 (0.67-1.63)	.84	1.02 (0.66-1.57)	.93
25-29 y	177/503 (35.0)	1 [Reference]	NA	1 [Reference]	NA	1 [Reference]	NA
30-34 y	190/651 (29.0)	0.76 (0.59-0.97)	.03	0.73 (0.57-0.96)	.03	0.76 (0.59-0.98)	.04
35-39 y	113/405 (28.0)	0.71 (0.54-0.95)	.02	0.66 (0.49-0.90)	.009	0.68 (0.50-0.91)	.01
≥40 y	25/100 (25.0)	0.61 (0.38-1.00)	.05	0.60 (0.35-1.02)	.06	0.61 (0.37-1.01)	.06
Japanese cedar IgE at age 4 y							
20-24 y	22/124 (18.0)	0.89 (0.53-1.49)	.66	0.84 (0.47-1.49)	.55	0.86 (0.50-1.51)	.61
25-29 y	98/503 (19.0)	1 [Reference]	NA	1 [Reference]	NA	1 [Reference]	NA
30-34 y	118/651 (18.0)	0.91 (0.68-1.23)	.56	1.02 (0.73-1.42)	.91	0.98 (0.71-1.36)	.92
35-39 y	87/405 (21.0)	1.13 (0.82-1.57)	.46	1.15 (0.79-1.67)	.45	1.14 (0.79-1.62)	.49
≥40 y	19/100 (19.0)	0.97 (0.56-1.68)	.91	1.23 (0.67-2.27)	.50	1.20 (0.66-2.18)	.54

Abbreviations: NA, not applicable; OR, odds ratio.

^a^ Multivariable logistic regression models were constructed by including confounding factors (ie, affiliated regional centers, maternal history of allergies, paternal history of allergies, maternal highest educational attainment, household income, child’s sex, birth weight, mode of delivery, number of siblings, household smoking during pregnancy, daycare, pets, and maternal body mass index), and the adjusted (multivariable model) ORs and their 95% CIs were calculated for each explanatory variable. For each explanatory variable, we calculated adjusted ORs from the multivariable model, as well as adjusted ORs obtained after multiple imputation of missing values, together with their 95% CIs.

For food allergy reaction at age 4 years, lower odds were also observed among children of mothers aged 30 to 34 years (OR, 0.91; 95% CI, 0.85-0.98), aged 35 to 39 years (OR, 0.81; 95% CI, 0.75-0.88), and aged 40 years or older (OR, 0.72; 95% CI, 0.61-0.84). Children of mothers aged 30 to 34 years (OR, 0.76; 95% CI, 0.59-0.98) and 35 to 39 years (OR, 0.68; 95% CI, 0.50-0.91) had lower odds of HDM sensitization, whereas no associations were found for Japanese cedar sensitization.

### Parental Age and Odds of Childhood Allergy at Ages 1, 2, and 4 Years

At age 1 year, children of parents both aged 35 years or older had lower odds of wheezing (OR, 0.89; 95% CI, 0.82-0.95) and food allergy reaction (OR, 0.90; 95% CI, 0.84-0.98) in Table 4. At age 4 years, similar inverse associations were observed for ever wheezing and food allergy reactions in children of parents both aged 35 years or older (Table 5).

**Table 4.** Association of Parental Age With Childhood Allergy at Ages 1 and 2 Years^a^

Outcome and parental age group	No. of children with outcome/total No. (%)	Univariable model		Multivariable model		Multiple imputation	
		OR (95% CI)	*P* value	OR (95% CI)	*P* value	OR (95% CI)	*P* value
Ever wheezing at age 1 y							
Mother ≤34 y, father ≤34 y	3768/20045 (19.0)	1 [Reference]	NA	1 [Reference]	NA	1 [Reference]	NA
Mother ≤34 y, father ≥35 y	1166/6234 (19.0)	0.99 (0.92-1.07)	.87	0.95 (0.88-1.03)	.25	0.96 (0.90-1.04)	.36
Mother ≥35 y, father ≤34 y	269/1577 (17.0)	0.89 (0.78-1.02)	.09	0.84 (0.72-0.96)	.01	0.83 (0.73-0.95)	.009
Mother ≥35 y, father ≥35 y	1231/6906 (18.0)	0.93 (0.87-1.01)	.07	0.88 (0.81-0.95)	.002	0.89 (0.82-0.95)	.002
Food allergy physician’s diagnosis at age 1 y							
Mother ≤34 y, father ≤34 y	1417/20157 (7.0)	1 [Reference]	NA	1 [Reference]	NA	1 [Reference]	NA
Mother ≤34 y, father ≥35 y	406/6260 (6.5)	0.91 (0.82-1.03)	.14	0.92 (0.82-1.05)	.22	0.92 (0.82-1.03)	.16
Mother ≥35 y, father ≤34 y	110/1586 (6.9)	0.99 (0.80-1.21)	.89	0.94 (0.76-1.17)	.61	0.97 (0.79-1.20)	.79
Mother ≥35 y, father ≥35 y	387/6939 (5.6)	0.78 (0.70-0.88)	<.001	0.75 (0.66-0.85)	<.001	0.77 (0.68-0.87)	<.001
Food allergy reactions at age 1 y							
Mother ≤34 y, father ≤34 y	3354/20124 (17.0)	1 [Reference]	NA	1 [Reference]	NA	1 [Reference]	NA
Mother ≤34 y, father ≥35 y	1000/6249 (16.0)	0.95 (0.88-1.03)	.21	0.97 (0.90-1.05)	.46	0.96 (0.89-1.04)	.35
Mother ≥35 y, father ≤34 y	254/1584 (16.0)	0.95 (0.83-1.09)	.52	0.94 (0.81-1.09)	.44	0.96 (0.83-1.11)	.54
Mother ≥35 y, father ≥35 y	1050/6927 (15.0)	0.90 (0.83-0.96)	.003	0.90 (0.83-0.97)	.009	0.90 (0.84-0.98)	.01
Atopic dermatitis physician’s diagnosis at age 1 y							
Mother ≤34 y, father ≤34 y	837/20157 (4.2)	1 [Reference]	NA	1 [Reference]	NA	1 [Reference]	NA
Mother ≤34 y, father ≥35 y	247/6260 (3.9)	0.95 (0.82-1.09)	.47	0.95 (0.81-1.11)	.52	0.95 (0.82-1.09)	.47
Mother ≥35 y, father ≤34 y	59/1586 (3.7)	0.90 (0.68-1.16)	.40	0.90 (0.68-1.20)	.50	0.87 (0.66-1.14)	.31
Mother ≥35 y, father ≥35 y	273/6939 (3.9)	0.94 (0.82-1.08)	.43	0.96 (0.83-1.13)	.69	0.95 (0.82-1.09)	.49
Eczema defined by International Study of Asthma and Allergies in Childhood at age 1 y							
Mother ≤34 y, father ≤34 y	3456/20144 (17.0)	1 [Reference]	NA	1 [Reference]	NA	1 [Reference]	NA
Mother ≤34 y, father ≥35 y	1139/6256 (18.0)	1.07 (1.00-1.16)	.06	1.08 (1.00-1.17)	.04	1.08 (1.00-1.17)	.04
Mother ≥35 y, father ≤34 y	314/1584 (20.0)	1.20 (1.05-1.36)	.007	1.23 (1.08-1.42)	.002	1.20 (1.04-1.36)	.008
Mother ≥35 y, father ≥35 y	1244/6936 (18.0)	1.05 (0.98-1.14)	.12	1.05 (0.97-1.14)	.25	1.06 (0.99-1.15)	.11
House dust mite IgE at age 2 y							
Mother ≤34 y, father ≤34 y	392/943 (42.0)	1 [Reference]	NA	1 [Reference]	NA	1 [Reference]	NA
Mother ≤34 y, father ≥35 y	129/335 (39.0)	0.88 (0.68-1.14)	.33	0.99 (0.76-1.30)	.96	0.92 (0.71-1.2)	.56
Mother ≥35 y, father ≤34 y	34/80 (43.0)	1.04 (0.66-1.65)	.87	1.09 (0.66-1.80)	.72	1.04 (0.65-1.67)	.88
Mother ≥35 y, father ≥35 y	188/425 (44.0)	1.12 (0.89-1.40)	.36	1.22 (0.94-1.58)	.13	1.19 (0.92-1.52)	.18
Japanese cedar IgE at age 2 y							
Mother ≤34 y, father ≤34 y	91/943 (9.7)	1 [Reference]	NA	1 [Reference]	NA	1 [Reference]	NA
Mother ≤34 y, father ≥35 y	26/335 (7.8)	0.79 (0.50-1.25)	.30	0.76 (0.47-1.22)	.25	0.74 (0.47-1.19)	.21
Mother ≥35 y, father ≤34 y	6/80 (7.5)	0.76 (0.32-1.79)	.53	0.82 (0.34-1.97)	.66	0.71 (0.30-1.72)	.45
Mother ≥35 y, father ≥35 y	42/425 (9.9)	1.03 (0.70-1.51)	.89	0.96 (0.62-1.48)	.84	0.98 (0.65-1.49)	.94

Abbreviations: NA, not applicable; OR, odds ratio.

^a^ Multivariable logistic regression models were constructed by including confounding factors (ie, affiliated regional centers, maternal history of allergies, paternal history of allergies, maternal highest educational attainment, household income, child’s sex, birth weight, mode of delivery, number of siblings, household smoking during pregnancy, daycare, pets, and maternal body mass index), and the adjusted (multivariable model) ORs and their CIs were calculated for each explanatory variable. For each explanatory variable, we calculated adjusted ORs from the multivariable model, as well as adjusted ORs obtained after multiple imputation of missing values, together with their 95% CIs.

**Table 5.** Association of Parental Age With Childhood Allergy at Age 4 Years^a^

Outcome and parental age group	No. of children with outcome/total No. (%)	Univariable model		Multivariable model		Multiple imputation	
		OR (95% CI)	*P* value	OR (95% CI)	*P* value	OR (95% CI)	*P* value
Current wheezing at age 4 y							
Mother ≤34 y, father ≤34 y	2942/20 112 (15.0)	1 [Reference]	NA	1 [Reference]	NA	1 [Reference]	NA
Mother ≤34 y, father ≥35 y	911/6243 (15.0)	1.00 (0.92-1.08)	.94	0.99 (0.90-1.08)	.83	1.00 (0.92-1.08)	.10
Mother ≥35 y, father ≤34 y	253/1582 (16.0)	1.12 (0.97-1.28)	.14	1.13 (0.97-1.31)	.11	1.09 (0.95-1.26)	.23
Mother ≥35 y, father ≥35 y	1001/6931 (14.0)	0.99 (0.91-1.06)	.71	0.98 (0.90-1.06)	.59	0.98 (0.90-1.06)	.67
Ever wheezing at age 4 y							
Mother ≤34 y, father ≤34 y	6028/20 111 (30.0)	1 [Reference]	NA	1 [Reference]	NA	1 [Reference]	NA
Mother ≤34 y, father ≥35 y	1793/6251 (29.0)	0.94 (0.89-1.00)	.05	0.93 (0.88-1.00)	.06	0.95 (0.89-1.01)	.12
Mother ≥35 y, father ≤34 y	455/1585 (29.0)	0.94 (0.84-1.05)	.29	0.94 (0.84-1.06)	.30	0.92 (0.82-1.03)	.15
Mother ≥35 y, father ≥35 y	1922/6917 (28.0)	0.90 (0.84-0.95)	<.001	0.91 (0.85-0.97)	.007	0.91 (0.85-0.97)	.004
Current asthma at age 4 y							
Mother ≤34 y, father ≤34 y	1801/20 157 (8.9)	1 [Reference]	NA	1 [Reference]	NA	1 [Reference]	NA
Mother ≤34 y, father ≥35 y	539/6260 (8.6)	0.98 (0.90-1.06)	.61	0.99 (0.90-1.08)	.83	0.98 (0.89-1.09)	.77
Mother ≥35 y, father ≤34 y	143/1586 (9.0)	0.96 (0.82-1.13)	.60	0.94 (0.79-1.12)	.47	1.00 (0.84-1.2)	.97
Mother ≥35 y, father ≥35 y	601/6939 (8.7)	0.97 (0.89-1.05)	.46	0.99 (0.9-1.08)	.77	0.99 (0.90-1.11)	.91
Ever asthma at age 4 y							
Mother ≤34 y, father ≤34 y	2427/20 088 (12.0)	1 [Reference]	NA	1 [Reference]	NA	1 [Reference]	NA
Mother ≤34 y, father ≥35 y	739/6241 (12.0)	0.98 (0.90-1.06)	.61	0.99 (0.9-1.08)	.83	0.99 (0.91-1.08)	.91
Mother ≥35 y, father ≤34 y	184/1582 (12.0)	0.96 (0.82-1.13)	.60	0.94 (0.79-1.12)	.47	0.94 (0.79-1.11)	.44
Mother ≥35 y, father ≥35 y	812/6913 (12.0)	0.97 (0.89-1.05)	.46	0.99 (0.9-1.08)	.77	0.99 (0.90-1.08)	.76
Current rhinitis at age 4 y							
Mother ≤34 y, father ≤34 y	1639/20 157 (8.1)	1 [Reference]	NA	1 [Reference]	NA	1 [Reference]	NA
Mother ≤34 y, father ≥35 y	518/6260 (8.3)	1.02 (0.92-1.13)	.72	1.09 (0.98-1.22)	.10	1.08 (0.97-1.20)	.14
Mother ≥35 y, father ≤34 y	119/1586 (7.5)	0.91 (0.76-1.12)	.38	0.98 (0.80-1.21)	.87	0.95 (0.79-1.16)	.64
Mother ≥35 y, father ≥35 y	516/6939 (7.4)	0.90 (0.82-1.01)	.07	1.01 (0.90-1.14)	.84	1.02 (0.91-1.14)	.73
Hay fever at age 4 y							
Mother ≤34 y, father ≤34 y	1934/20 157 (9.6)	1 [Reference]	NA	1 [Reference]	NA	1 [Reference]	NA
Mother ≤34 y, father ≥35 y	597/6260 (9.5)	0.99 (0.90-1.09)	.90	1.06(0.96-1.19)	.23	1.05 (0.95-1.16)	.31
Mother ≥35 y, father ≤34 y	155/1586 (9.8)	1.02 (0.86-1.21)	.82	1.06 (0.89-1.28)	.49	1.06 (0.90-1.27)	.48
Mother ≥35 y, father ≥35 y	629/6939 (9.1)	0.94 (0.85-1.03)	.19	1.04 (0.94-1.16)	.44	1.05 (0.95-1.16)	.33
Food allergy physician’s diagnosis at age 4 y							
Mother ≤34 y, father ≤34 y	1211/20 157 (6.0)	1 [Reference]	NA	1 [Reference]	NA	1 [Reference]	NA
Mother ≤34 y, father ≥35 y	364/6260 (5.8)	0.97 (0.85-1.09)	.57	1.02 (0.90-1.16)	.73	1.00 (0.88-1.13)	.95
Mother ≥35 y, father ≤34 y	86/1586 (5.4)	0.90 (0.72-1.13)	.34	0.93 (0.74-1.19)	.57	0.90 (0.73-1.14)	.41
Mother ≥35 y, father ≥35 y	356/6939 (5.1)	0.84 (0.75-0.95)	.007	0.89 (0.78-1.02)	.09	0.89 (0.78-1)	.06
Food allergy reactions at age 4 y							
Mother ≤34 y, father ≤34 y	4004/20 157 (20.0)	1 [Reference]	NA	1 [Reference]	NA	1 [Reference]	NA
Mother ≤34 y, father ≥35 y	1210/6260 (19.0)	0.97 (0.90-1.04)	.35	1.01 (0.93-1.08)	.88	0.99 (0.92-1.07)	.83
Mother ≥35 y, father ≤34 y	280/1586 (18.0)	0.86 (0.76-0.99)	.03	0.88 (0.76-1.01)	.08	0.87 (0.76-1.00)	.04
Mother ≥35 y, father ≥35 y	1165/6939 (17.0)	0.81 (0.76-0.88)	<.001	0.827 (0.76-0.90)	<.001	0.84 (0.78-0.90)	<.001
Atopic dermatitis physician’s diagnosis at age 4 y							
Mother ≤34 y, father ≤34 y	1685/20 157 (8.4)	1 [Reference]	NA	1 [Reference]	NA	1 [Reference]	NA
Mother ≤34 y, father ≥35 y	510/6260 (8.1)	0.97 (0.88-1.08)	.60	1.01 (0.90-1.14)	.79	0.98 (0.88-1.08)	.64
Mother ≥35 y, father ≤34 y	142/1586 (9.0)	1.08 (0.90-1.28)	.41	1.09 (0.90-1.32)	.34	1.06 (0.89-1.27)	.51
Mother ≥35 y, father ≥35 y	505/6939 (7.3)	0.86 (0.78-0.95)	.004	0.90 (0.79-1.00)	.05	0.89 (0.79-0.99)	.02
Eczema defined by International Study of Asthma and Allergies in Childhood at age 4 y							
Mother ≤34 y, father ≤34 y	2704/20 118 (13.0)	1 [Reference]	NA	1 [Reference]	NA	1 [Reference]	NA
Mother ≤34 y, father ≥35 y	866/6248 (14.0)	1.04 (0.95-1.13)	.40	1.05 (0.96-1.15)	.26	1.04 (0.95-1.13)	.37
Mother ≥35 y, father ≤34 y	242/1581 (15.0)	1.16 (1.01-1.34)	.04	1.19 (1.02-1.38)	.02	1.16 (1.00-1.34)	.05
Mother ≥35 y, father ≥35 y	930/6931 (13.0)	1.00 (0.92-1.08)	.96	1.01 (0.92-1.11)	.78	1.01 (0.93-1.09)	.82
House dust mite IgE at age 4 y							
Mother ≤34 y, father ≤34 y	310/943 (33.0)	1 [Reference]	NA	1 [Reference]	NA	1 [Reference]	NA
Mother ≤34 y, father ≥35 y	101/335 (30.0)	0.88 (0.67-1.15)	.36	0.92 (0.69-1.23)	.57	0.92 (0.70-1.22)	.58
Mother ≥35 y, father ≤34 y	22/80 (28.0)	0.77 (0.47-1.28)	.33	0.61 (0.35-1.08)	.09	0.71 (0.42-1.20)	.20
Mother ≥35 y, father ≥35 y	116/425 (27.0)	0.76 (0.59-0.99)	.04	0.79 (0.60-1.05)	.11	0.77 (0.59-1.02)	.07
Japanese cedar IgE at age 4 y							
Mother ≤34 y, father ≤34 y	173/943 (18.0)	1 [Reference]	NA	1 [Reference]	NA	1 [Reference]	NA
Mother ≤34 y, father ≥35 y	65/335 (19.0)	1.07 (0.78-1.48)	.67	1.16 (0.82-1.63)	.40	1.11 (0.79-1.55)	.56
Mother ≥35 y, father ≤34 y	17/80 (21.0)	1.20 (0.68-2.10)	.52	0.99 (0.52-1.86)	.97	1.08 (0.60-1.95)	.79
Mother ≥35 y, father ≥35 y	89/425 (21.0)	1.17 (0.89-1.57)	.26	1.27 (0.91-1.77)	.15	1.23 (0.90-1.68)	.21

Abbreviations: NA, not applicable; OR, odds ratio.

^a^ Multivariable logistic regression models were constructed by including confounding factors (ie, affiliated regional centers, maternal history of allergies, paternal history of allergies, maternal highest educational attainment, household income, child’s sex, birth weight, mode of delivery, number of siblings, household smoking during pregnancy, daycare, pets, and maternal body mass index), and the adjusted (multivariable model) ORs and their CIs were calculated for each explanatory variable. For each explanatory variable, we calculated adjusted ORs from the multivariable model, as well as adjusted ORs obtained after multiple imputation of missing values, together with their 95% CIs.

## Discussion

In this cohort study, we observed that even after adjusting for factors such as parental educational level, household income, number of siblings, and family history of allergy, children of older mothers had lower prevalence of food allergy outcomes. On the other hand, children of younger mothers (20-24 years) had higher odds of wheezing at the age of 2 and 4 years. Our initial hypothesis was that aging might contribute to epigenetic changes related to allergic diseases in children. However, the observed association with food allergy outcomes was unexpected. At age 1 year, both younger and older maternal age groups demonstrated different odds of reported food allergy diagnoses or reactions compared with the reference group, indicating a nonlinear association that warrants further investigation. Interestingly, children of mothers aged 20 to 24 years showed a slightly lower odds of food allergy compared with those of mothers aged 25 to 29 years. The reasons for this nonlinear pattern are not fully clear and cannot be definitively determined from this observational study. Possible explanations include differences in maternal lifestyle, environmental exposures, or other unmeasured confounding factors that may vary across age groups. This nonlinear pattern suggests that the association between maternal age and food allergy in early infancy may be influenced by additional factors, such as differences in feeding practices, diagnostic awareness, or health care–seeking behavior. These findings also indicate that maternal age may be associated differently with food allergy depending on the child’s age and developmental stage. Further longitudinal research is warranted to clarify these age-specific patterns.

Several potential mechanisms may explain why advanced parental age was associated with lower odds of food allergy. One possibility is greater economic and social stability for older parents. Older parents are more likely to have established careers and stable lifestyle, which may enable them to provide their children with more favorable educational and living environments. Another factor may be greater psychological resilience in parenting. With increased life experience, older parents may approach child-rearing with greater emotional stability, which could, in turn, contribute to the emotional well-being of their children. Additionally, older parents may have more opportunities to interact with individuals across various age groups, fostering a broader perspective. Furthermore, prolonged exposure to health-related information and accumulated life experience may enhance parental health literacy (HL), which could contribute to the reduced incidence of food allergy observed in this study.

A previous study^26^ found that mothers’ HL is significantly associated with early childhood allergy prevention behaviors. Specifically, lower HL is associated with increased allergen-avoiding behaviors and a decreased likelihood of exclusive breastfeeding. This suggests that improved HL could enhance food allergy management by promoting recommended behaviors, such as breastfeeding, while reducing unnecessary allergen avoidance. Therefore, enhancing mothers’ HL may improve food allergy management practices in families. Another previous study^27^ highlighted that HL is crucial for parents to understand and apply recommendations for early childhood allergy prevention. Pediatricians noted the importance of assessing parental information levels to ensure effective communication about allergy management. However, they lacked formal HL screening methods, relying instead on intuition. This indicates that enhancing HL among parents can improve their ability to manage food allergies effectively, emphasizing the need for HL-sensitive consultations in pediatric care.

Several randomized clinical trials^28,29,30,31,32,33^ on prevention are emerging. On the basis of the dual allergen exposure hypothesis, early intervention for eczema and early introduction of allergenic foods are key strategies.^34^ To translate these evidence-based approaches into public health practice, it is essential to provide information from the prenatal period.^35,36^ Enhancing parental HL will likely play a crucial role in the successful implementation of these preventive measures. Allergy education initiatives targeting school children and staff may further support preventive strategies.^37^ When providing information, it is important to consider the background of parents. On the basis of the findings of this study, particular attention should be given to ensuring that younger parents receive adequate information.

Additionally, we observed an inverse association between advanced maternal age and sensitization to HDMs, a finding not previously reported to our knowledge. In Japanese school-aged children in Tokyo, HDM sensitization is highly prevalent, with more than one-half of the general population exhibiting sensitization.^38^ Given that the concentration of HDMs in the environment is thought to be associated with HDM sensitization in children, one possible explanation for the lower sensitization observed in children of older mothers would be that these mothers may have greater knowledge about HDMs and may have implemented more effective environmental control measures in their homes.

### Strengths and Limitations

The strength of this study lies in the use of large-scale data from a national birth cohort, providing high external validity and reflecting general population community data across Japan. However, several limitations should be noted. First, the inverse associations between advanced parental age and childhood allergic outcomes should be interpreted cautiously. Although we adjusted for a wide range of sociodemographic and perinatal covariates, residual confounding cannot be excluded. Potential explanatory factors include parental health behaviors, feeding and breastfeeding practices, prenatal nutrition, vaccination exposure, and early-life antibiotic or acid-suppressant use. Because this study adhered to a predefined analysis plan and detailed longitudinal exposure data up to age 4 years were unavailable, these variables were not included in the multivariable models. Future studies incorporating comprehensive, time-varying data are needed to clarify whether the observed associations reflect true protective effects or residual confounding. This study was based on specific hypotheses regarding the impact of parental age on childhood allergic diseases. Although we observed a protective association of older parental age, these findings were somewhat unexpected from a biological standpoint. As an observational epidemiological study, it cannot elucidate biological mechanisms or establish causality. Further mechanistic research integrating epidemiological, molecular, and experimental approaches will be needed to clarify underlying biological pathways, including genetic and epigenetic factors related to food allergy.^39^

Second, this study was conducted in a general population rather than a hospital-based setting, which means that linkage with detailed hospital data was not possible. Detailed clinical data from hospitals, such as treatment history, prescription records, and disease severity, were not available in this cohort. Therefore, we were unable to evaluate medication use or severity of allergic diseases, which we acknowledge as a limitation of this study. In addition, the prevalence of food allergy was assessed solely by questionnaire, which may lead to overestimation. Although this method has been used in our previous reports and allows for large-scale data collection, the potential for overestimation should be taken into account when interpreting the results.

## Conclusions

In conclusion, this study addressed that advanced maternal age was associated with a lower prevalence of food allergies, wheezing, and asthma in children, suggesting a protective effect. This may be attributed to greater economic stability, HL, and better environmental control measures among older parents. The study also highlighted the association between maternal age and reduced HDM sensitization in children. Understanding the influence of parental age on allergic disease risk may inform targeted prevention strategies and public health policies aimed at reducing the burden of pediatric allergies. Further research is needed to elucidate the underlying mechanisms behind these associations.

## Notes

Supplement 1. eMethods. eTable. The prevalence of allergic outcomes eFigure. Flow chart of the study

Supplement 2. Nonauthor Collaborators. Japan Environment and Children’s Study (JECS) Group

Supplement 3. Data Sharing Statement
