# Supplementary material for: Parental Age and Childhood Allergy Risk
Source: JAMA Netw Open. 2026 Jan 20;9(1):e2554694. doi: 10.1001/jamanetworkopen.2025.54694 (PMC12820740; doi:10.1001/jamanetworkopen.2025.54694)
Supplement: Supplement 1. — eMethods. eTable. The prevalence of allergic outcomes eFigure. Flow chart of the study [file jamanetwopen-e2554694-s001.pdf]

Supplemental Online Content

Yamamoto-Hanada K, Harama D, Sato M, et al; Japan Environment and Children's Study (JECS) Group. Parental age and childhood allergy risk. *JAMA Netw Open*. 2026;9(1):e2554694. doi:10.1001/jamanetworkopen.2025.54694

eMethods.

eTable. The prevalence of allergic outcomes

eFigure. Flow chart of the study

This supplemental material has been provided by the authors to give readers additional information about their work.

© 2026 Yamamoto-Hanada K et al. *JAMA Network Open*.

eMethods

Detailed statistical procedures

Multiple imputation was conducted using the chained equations algorithm implemented in the R package mice (version 3.16.0). Twenty imputed datasets (m = 20) were generated, each with 20 iterations. Prior to imputation, we confirmed that none of the variables included in the analysis had more than 20% missingness. To ensure consistency with the study design, identifiers and outcome variables were excluded from both imputation and prediction. This was implemented by modifying the predictor matrix and imputation mask accordingly.

For continuous variables, predictive mean matching was used; for binary variables, logistic regression was used; and for ordinal variables, proportional odds models were applied. All variables included in the multivariable models were also included as predictors in the imputation model. Estimates from the 20 datasets were combined using Rubin's rules.

To account for potential heterogeneity among the 15 regional centers, study site was treated as a categorical covariate in all multivariable models. Further details regarding the treatment of site-level variation are described here.

Multivariable logistic regression models using imputed datasets included the same set of confounding factors: affiliated regional centers, maternal history of allergies, paternal history of allergies, maternal educational attainment, household income, child's sex, birth weight, mode of delivery, number of siblings, household smoking during pregnancy, daycare attendance, pet ownership, and maternal BMI. The adjusted odds ratios (OR_MI) and corresponding 95% confidence intervals were calculated for each explanatory variable.

For reference, p-values were provided in the tables were two-sided, with emphasis placed on the magnitude of the estimates and whether the 95% CIs included 1. Accordingly, p-values reported in the tables are provided for reference only and should be interpreted as descriptive indicators rather than formal tests of statistical significance. Because this study was exploratory and aimed to identify potential associations rather than to test predefined hypotheses, no corrections for multiple comparisons (e.g., false discovery rate adjustment) were applied, as the analyses were intended to generate hypotheses for future confirmatory research.

Analyses using IgE as the outcome variable were performed only among children with available IgE measurements, using the same procedures described above.

© 2026 Yamamoto-Hanada K et al. *JAMA Network Open*.

As for treatment of site-level variation, In this cohort study spanning 15 regional centers, participants were recruited and assessed within distinct institutional settings. Each center operates under its own study staff, catchment population, and local healthcare infrastructure, which may contribute to site-level heterogeneity in both exposures and outcomes.

To account for potential confounding by study site, all multivariable models included “center” as a categorical covariate. This approach adjusts for systematic differences across sites without modeling site-level effects as random or correlated residuals. While we acknowledge that intra-site correlation may violate the assumption of independent and identically distributed observations, we chose not to implement cluster-robust standard errors in this analysis. This decision was made to maintain consistency with prior publications from the same cohort, which have uniformly applied conventional regression models with site adjustment via fixed effects.

We acknowledge that cluster-robust estimators (e.g., Huber–White sandwich variance) represent an alternative approach to handling intra-cluster correlation. However, given the relatively large number of centers and the inclusion of site as a fixed covariate, we believe our current strategy provides a reasonable balance between interpretability, comparability, and statistical control.

Sensitivity analyses exploring center-level variation (e.g., stratified models or center-by-exposure interaction terms) may be considered in future work.

© 2026 Yamamoto-Hanada K et al. *JAMA Network Open*.

eTable. The prevalence of allergic outcomes

Category	N(%)	Proportion [95%CI by Clopper-Pearson]
Ever wheezing at 1 year old		
No	28,328 (81.1%)	
Yes	6,434 (18.4%)	18.4 [95%CI: 18.0 - 18.8] %
(Missing)	180 (0.5%)	
Food allergy doctor's diagnosis at 1 year old		
No	32,622 (93.4%)	
Yes	2,320 (6.6%)	6.6 [95%CI: 6.4 - 6.9] %
Food allergy reactions at 1 year old		
No	29,226 (83.6%)	
Yes	5,658 (16.2%)	16.2 [95%CI: 15.8 - 16.6] %
(Missing)	58 (0.2%)	
Atopic dermatitis doctor's diagnosis at 1 year old		
No	33,526 (95.9%)	
Yes	1,416 (4.1%)	4.1 [95%CI: 3.8 - 4.3] %
Eczema defined by ISAAC at 1 year old		
No	28,767 (82.3%)	
Yes	6,153 (17.6%)	17.6 [95%CI: 17.2 - 18.0] %
(Missing)	22 (0.1%)	
House dust mite IgE at 2 years old		
No	1,165 (3.3%)	
Yes	826 (2.4%)	2.4 [95%CI: 2.2 - 2.5] %
(Missing)	32,951 (94.3%)	
Japanese cedar IgE at 2 years old		
No	1,807 (5.2%)	
Yes	184 (0.5%)	0.5 [95%CI: 0.5 - 0.6] %
(Missing)	32,951 (94.3%)	
Current wheezing at 4 years old		
No	29,761 (85.2%)	
Yes	5,107 (14.6%)	14.6 [95%CI: 14.2 - 15.0] %
(Missing)	74 (0.2%)	
Ever wheezing at 4 years old		

© 2026 Yamamoto-Hanada K et al. *JAMA Network Open.*

No	24,666 (70.6%)	
Yes	10,198 (29.2%)	29.2 [95%CI: 28.7 - 29.7] %
(Missing)	78 (0.2%)	
Current asthma at 4 years old		
No	31,858 (91.2%)	
Yes	3,084 (8.8%)	8.8 [95%CI: 8.5 - 9.1] %
Ever asthma at 4 years old		
No	30,662 (87.8%)	
Yes	4,162 (11.9%)	11.9 [95%CI: 11.6 - 12.3] %
(Missing)	118 (0.3%)	
Current rhinitis at 4 years old		
No	32,150 (92.0%)	
Yes	2,792 (8.0%)	8.0 [95%CI: 7.7 - 8.3] %
Hay fever at 4 years old		
No	31,627 (90.5%)	
Yes	3,315 (9.5%)	9.5 [95%CI: 9.2 - 9.8] %
Food allergy doctor's diagnosis at 4 years old		
No	32,925 (94.2%)	
Yes	2,017 (5.8%)	5.8 [95%CI: 5.5 - 6.0] %
Food allergy reactions at 4 years old		
No	28,283 (80.9%)	
Yes	6,659 (19.1%)	19.1 [95%CI: 18.6 - 19.5] %
Atopic dermatitis doctor's diagnosis at 4 years old		
No	32,100 (91.9%)	
Yes	2,842 (8.1%)	8.1 [95%CI: 7.8 - 8.4] %
Eczema defined by ISAAC at 4 years old		
No	30,136 (86.2%)	
Yes	4,742 (13.6%)	13.6 [95%CI: 13.2 - 13.9] %
(Missing)	64 (0.2%)	
House dust mite IgE at 4 years old		
No	1,270 (3.6%)	
Yes	570 (1.6%)	1.6 [95%CI: 1.5 - 1.8] %
(Missing)	33,102 (94.7%)	
Japanese cedar IgE at 4years old		
No	1,486 (4.3%)	

© 2026 Yamamoto-Hanada K et al. *JAMA Network Open.*

Yes	354 (1.0%)	1.0 [95%CI: 0.9 - 1.1] %
(Missing)	33,102 (94.7%)	

© 2026 Yamamoto-Hanada K et al. *JAMA Network Open*.

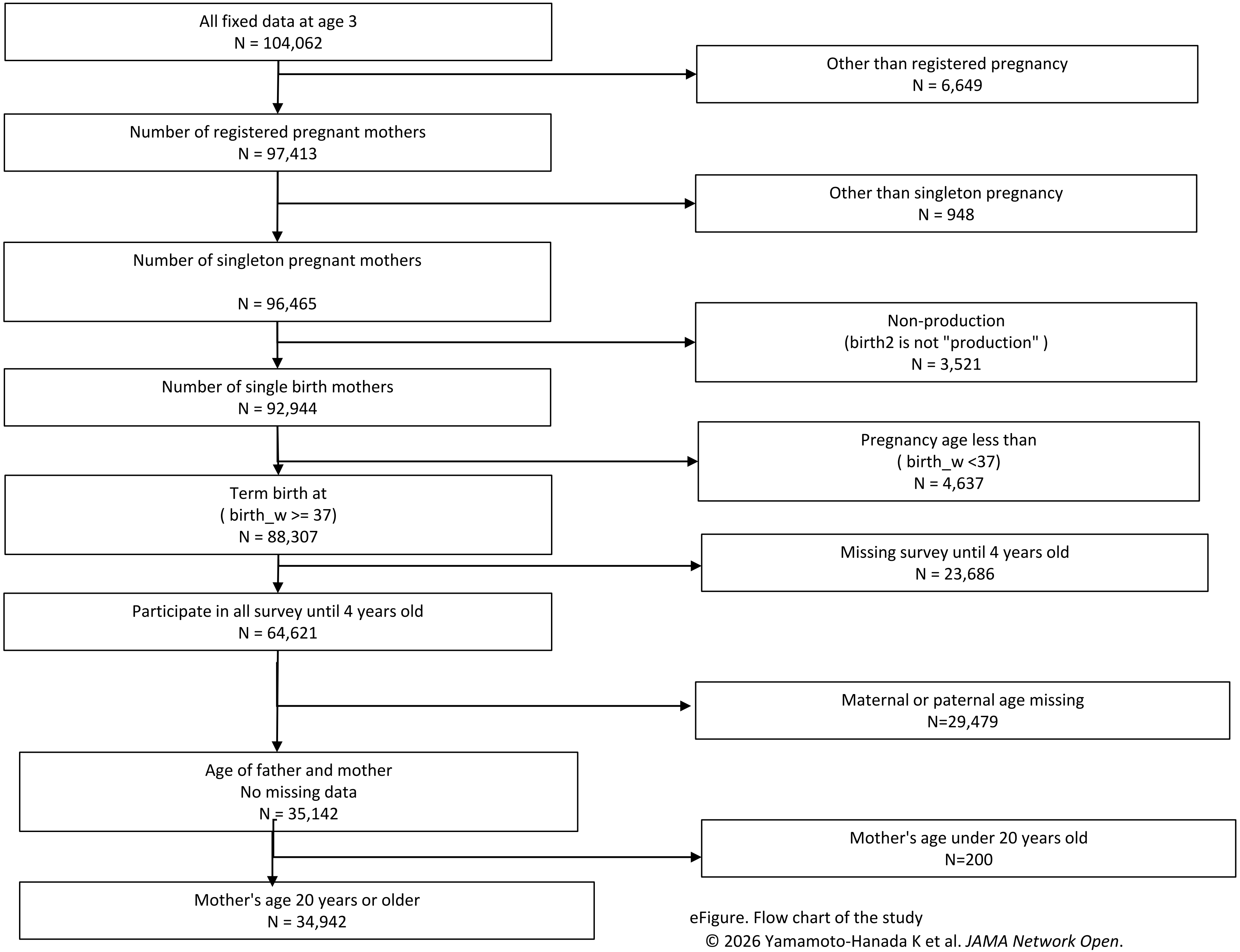

eFigure. Flow chart of the study

© 2026 Yamamoto-Hanada K et al. *JAMA Network Open*.
