# Supplementary material for: Parental Age and Childhood Allergy Risk
Source: JAMA Netw Open. 2026 Jan 20;9(1):e2554694. doi: 10.1001/jamanetworkopen.2025.54694 (PMC12820740; doi:10.1001/jamanetworkopen.2025.54694)
Supplement: Supplement 2. — Nonauthor Collaborators. Japan Environment and Children’s Study (JECS) Group [file jamanetwopen-e2554694-s002.pdf]

*Group Name(s): Japan Environment and Children's Study (JECS) Group

*First Name and Middle Initial(s)	*Last Name	Academic Degrees	Institution	Location (city, state/province, country)	Role or Contribution, eg, chair, principal investigator
Michihiro	Kamijima	MD, PhD	Nagoya City University	Nagoya, Japan	Primay-Investigator
Shin	Yamazaki	MD, PhD	National Institute for Environmental	Tsukuba, Japan	Co-Investigator
Reiko	Kishi	MD, PhD	Hokkaido University	Sapporo, Japan	Co-Investigator
Chiharu	Ota	MD, PhD	Tohoku University	Sendai, Japan	Co-Investigator
Koichi	Hashimoto	MD, PhD	Fukushima Medical University	Fukushima, Japan	Co-Investigator
Chisato	Mori	MD, PhD	Chiba University	Chiba, Japan	Co-Investigator
Shuichi	Ito	MD, PhD	Yokohama City University	Yokohama, Japan	Co-Investigator
Ryoji	Shinohara	MD, PhD	University of Yamanashi	Chuo, Japan	Co-Investigator
Hidekuni	Inadera	MD, PhD	University of Toyama	Toyama, Japan	Co-Investigator
Takeo	Nakayama	MD, PhD	Kyoto University	Kyoto, Japan	Co-Investigator
Ryo	Kawasaki	MD, PhD	Osaka University	Suita, Japan	Co-Investigator
Yasuhiro	Takeshima	MD, PhD	Hyogo Medical University	Nishinomiya, Japan	Co-Investigator
Seiji	Kageyama	MD, PhD	Tottori University	Yonago, Japan	Co-Investigator
Narufumi	Suganuma	MD, PhD	Kochi University	Nankoku, Japan	Co-Investigator
Shoichi	Ohga	MD, PhD	Kyushu University	Fukuoka, Japan	Co-Investigator
Takahiko	Katoh	MD, PhD	Kumamoto University	Kumamoto, Japan	Co-Investigator
