# Supplementary material for: Parental Age and Childhood Allergy Risk
Source: JAMA Netw Open. 2026 Jan 20;9(1):e2554694. doi: 10.1001/jamanetworkopen.2025.54694 (PMC12820740; doi:10.1001/jamanetworkopen.2025.54694)
Supplement: Supplement 3. — Data Sharing Statement [file jamanetwopen-e2554694-s003.pdf]

Data Sharing Statement

Yamamoto-Hanada. Parental Age and Childhood Allergy Risk. *JAMA Netw Open*. Published January 20, 2026. doi:10.1001/jamanetworkopen.2025.54694

Data
Data available: No

Additional Information
Explanation for why data not available: The data sharing is not available now.
